# Draft Genome Sequence of *Bacillus fengqiuensis* FJAT-14578, Isolated from a Soil Sample in China

**DOI:** 10.1128/genomeA.01126-14

**Published:** 2014-11-06

**Authors:** Guo-Hong Liu, Bo Liu, Jian-Yang Tang, Jian-Mei Che, Yu-Jing Zhu, Ming-Xing Su, Jie-Ping Wang, Qian-Qian Chen

**Affiliations:** Agricultural Bio-resource Institute, Fujian Academy of Agricultural Sciences, Fuzhou, Fujian, China

## Abstract

Here, we report the first high-quality draft genome sequence of *Bacillus fengqiuensis* FJAT-14578, isolated from a soil sample collected from China. The genome size was 5,569,389 bp, with a 40.93 mol% G+C content. The number of tRNAs was 69 and of rRNAs was 10 (5S, 16S, and 23S).

## GENOME ANNOUNCEMENT

*Bacillus fengqiuensis* FJAT-14578 (16S rRNA GenBank accession no. JX262265.1), a Gram-positive endospore-forming aerobic bacterium, was isolated from a soil sample in China. Recently, this species was first found and reported by Zhao et al. (1) and was isolated from a typical sandy loam soil under long-term NPK fertilization in northern China. One genome assembly for the strain *B*. *fengqiuensis* FJAT-14578 was already available at GenBank, but no other genome sequence of this *B*. *fengqiuensis* strain was found in the database. Strain FJAT-14578 was found to be slightly facultatively alkaliphilic, with optimum growth occurring in 0% to 2.0% (wt/vol) NaCl and at pH 7.0. Colonies were red pigmented and flat, had circular/slightly irregular margins, and were 2 to 3 mm in diameter after incubation for 2 days at 30°C on nutrient agar (NA). Cells grew well on NA and Luria-Bertani (LB) agar, but showed weak growth on tryptic soy agar (TSA). The optimum temperature for growth was 30 to 35°C.

Genome sequencing was performed on an Illumina HiSeq 2000 sequencer (Illumina, Inc.) by generating paired-end libraries, and sequencing was performed at the Beijing Genomics Institute (BGI) (Shenzhen, China). Paired-end reads were *de novo* assembled using SOAPdenovo v. 1.05 (2) and gaps were filled manually. Gene prediction was performed using Glimmer v. 3.02 (3), and rRNA and tRNA were identified using RNAmmer (4) and tRNAscan-SE 1.3.1 (5), respectively. The genome sequence was annotated and functional annotation was performed using Cluster of Orthologous Groups (COG) and the Kyoto Encyclopedia of Genes and Genomes (KEGG) (6).

Using an Illumina HiSeq 2000 sequencing platform, we produced 1,089 Mb of data for a sample of FJAT-14578. Based on the assembly result of this sample of FJAT-14578, we found that the genome size was 5,569,389 bp, with a 40.93 mol% G+C content. The numbers of scaffolds and contigs were 37 and 43, respectively. From the genome analysis results of FJAT-14578, we found that the genome contained 5,684 genes, and the total length of genes was 4,553,907 bp, which makes up 81.77% of the genome. The number of tandem repeat sequences was 195, and the total length of tandem repeat sequences was 13,948 bp, which makes up 0.2504% of the genome. The number of minisatellite DNAs was 126, the number of microsatellite DNAs was 21, the number of tRNAs was 69, and the number of rRNAs was 10 (5S, 16S, and 23S). According to the KEGG and COG analyses, the KEGG pathway divides the biological pathways into eight main parts, and each part includes several subparts. The COG database divided them into 20 parts by their functions. In addition, 55 hypothetical proteins and 287 unknown functions of the genome sequence were identified.

### Nucleotide sequence accession number.

The draft genome sequence of *B. fengqiuensis* FJAT-14578 has been deposited at DDBJ/EMBL/GenBank under the accession no. AYSE00000000. The version described in this paper is the first version.
